# Understanding the Impact of a New Approach to the Safeguarding of Children at Risk: An Evaluation Protocol

**DOI:** 10.5334/ijic.5980

**Published:** 2022-11-11

**Authors:** Ruta Buivydaite, Apostolos Tsiachristas, Steve Thomas, Hannah Farncombe, Rafael Perera-Salazar, Ray Fitzpatrick, Charles Vincent

**Affiliations:** 1 Department of Experimental Psychology, University of Oxford, Radcliffe House, Radcliffe Observatory Quarter, Woodstock Road, Oxford, OX2 6GG, UK; 2 Health Economics Research Centre, Nuffield Department of Population Health, University of Oxford, Richard Doll Building, Old Road Campus, OxfordOX3 7LF, UK; 3 Oxfordshire Children Social Services, County Hall, New Road, Oxford, OX1 1ND, UK; 4 Medical Statistics Group, Nuffield Department of Primary Care Health Sciences, University of Oxford, Oxford, UK; 5 Health Services Research Unit, Nuffield Department of Population Health, University of Oxford, Oxford, UK

**Keywords:** children social care, service change, complex intervention, evaluation protocol

## Abstract

**Introduction:**

Child Safeguarding Services intervene in situations where a child is at risk of serious emotional or physical harm. The response will vary according to the level of risk, but in serious cases, a child may need to be removed from danger and cared for by foster parents either temporarily or permanently. The number of children being taken into care has increased markedly in recent years in the United Kingdom. Oxfordshire County Council (OCC) is implementing a new approach to the welfare of children (Family Solutions Plus; FSP) in which the focus is to support the whole family and ideally reduce the need for foster care.

In this paper, we describe a proposed programme of evaluation to examine the impact of FSP on the time children are in contact with services, the nature of the support provided, experience of children and families, the experience of staff, and longer term outcomes for children, particularly whether they remain within the family or need to be cared for outside the home.

**Methods and analysis:**

A mixed methods approach will be taken in an observational retrospective study of children’s social care services. Quantitative research will include descriptive analysis on data routinely collected by OCC, examining the effect on time spent in services, outcomes for children and how these outcomes are mediated by family characteristics and circumstances. Qualitative research will be carried out using individual interviews and focus groups with children, families and staff in the teams providing family safeguarding services to capture their experiences with the new model.

**Ethics and dissemination:**

This project has been registered with the OCC as a service evaluation. The qualitative studies will seek ethical approval from Oxford University Ethics Committee. A local data sharing agreement will govern the transfer of quantitative data. Results will be disseminated through newsletters, community forums, professional publications and conference presentations to national and international audiences.

## Introduction

Child Safeguarding Services intervene in situations where a child is at risk of serious emotional or physical harm [1]. The response will vary according to the level of risk, but in serious cases a child may need to be removed from immediate danger and cared for by foster parents either temporarily or permanently. In Britain, some estimates suggest that up to 30% of children have experienced some form of abuse or neglect from their caregivers at some point in their lives [2]. In England, local councils run child safeguarding services, which assure the safety and well-being of children.

Placing a child in care protects them and ensures their safety in the short-term. However, removing the child from their family has significant adverse effects on their well-being in the longer-term [3,4,5,6,7]. Schneider and colleagues [4] found that if the child spends longer than 6 weeks in foster care, their mental health and development are affected. Social services therefore face a dilemma: they have to protect a child from harm in their home, while simultaneously recognising the detrimental effect of removing a child from their family.

In this paper, we set out a protocol for the evaluation of a major change in safeguarding services implemented by Oxfordshire County Council (OCC) in November 2020. We describe the nature of this change and the methods that we will use to monitor changes in services and how we will evaluate the impact on families, staff and the use of services.

### Child Safeguarding in England

The Children Act 1989 placed a duty on the local authority to provide services to children in need in their area. The Children Act 2004 further enhanced the role of local authorities, requiring them to undertake enquiries if they believe a child has suffered or is likely to suffer significant harm, and also requiring them to work with partner agencies such as the National Health Service (NHS) and police.

Child safeguarding services have evolved considerably since 2004 with introduction of standardised assessment frameworks and promotion of multi-agency collaboration [8,9]. However, many authorities remained concerned that wider problems in the family, such as mental health and substance abuse, were not being given sufficient attention and were impeding efforts to support children [10]. A need was identified for a new holistic approach to child safeguarding, delivering services on a whole family basis, prompting the development of a new approach to family safeguarding by Hertfordshire County Council.

The new approach was first developed and trialled by Hertfordshire County Council in 2015, where it delivered improved outcomes for children and their families whilst also significantly reducing demands and costs for the county [11]. This approach represented a significant shift from child focused services to an integrated family-oriented model of practice. The revised model concentrates on the care of both child and adult family members. This is a unique aspect because the health and well-being of adult family members impacts the welfare of a child [12]. Thus, the model is reliant on the active engagement of families.

This safeguarding approach therefore represents a move towards more integrated care in a number of respects. First, the support provided encourages the positive and active engagement of the whole family. Second, services to the family are brought within one umbrella service, rather than the fragmented method of offering single interventions to single family members. Third, the approach addresses the different facets of child and family well-being in a holistic manner, addressing parenting, education, mental health and living conditions within the programme. Finally, the programme and others that followed aims to improve communication and coordination with the family and between services [13,14,15].

In 2017, the Department for Education (DfE) commissioned an evaluation of the Hertfordshire approach to family safeguarding which reported a 38% reduction in care proceedings, 50% reduction in the use of child protection plans, over 50% reduction in emergency hospital admission and police call outs to the families concerned and cost savings of £2.6million in the first year. Staff across all disciplines reported feeling more confident and less stressed, and positive effects were reported on recruitment and retention.

The Hertfordshire approach has been evaluated as being effective and received praise from Ofsted.^1^ This model has been replicated by at least twelve other English local authorities [11]. Despite having been evaluated as effective, the evaluations conducted so far are primarily qualitative in nature and have made only limited analysis of statutory data sources [9,17,18]. In addition, the majority of evaluations are done internally and so there is limited access to the data or analyses conducted [19,20,21].

### Family Solutions Plus: a new approach in Oxfordshire

In Oxfordshire, the previous children’s safeguarding service focussed on the welfare of the child at risk rather than the family as a whole. The service included Children and Family Assessment Central Teams (CAFAT) who were responsible for assessment, and separate Family Solution Services (FSS) teams that delivered social care for the child, together with Early Help (EH) teams. CAFAT and FSS provide statutory services, handling some of the most challenging parts of children’s social care, and this was reflected in very low staff retention rates.

OCC and partner agencies made a commitment to address the vulnerabilities in families that lead to breakdown, to provide stronger and more wide-ranging support and to reduce the need for children to be taken into care. After reviewing models of national best practice, OCC developed a new approach to safeguarding: Family Solutions Plus (FSP) launched in November 2020.

**Figure 1:**
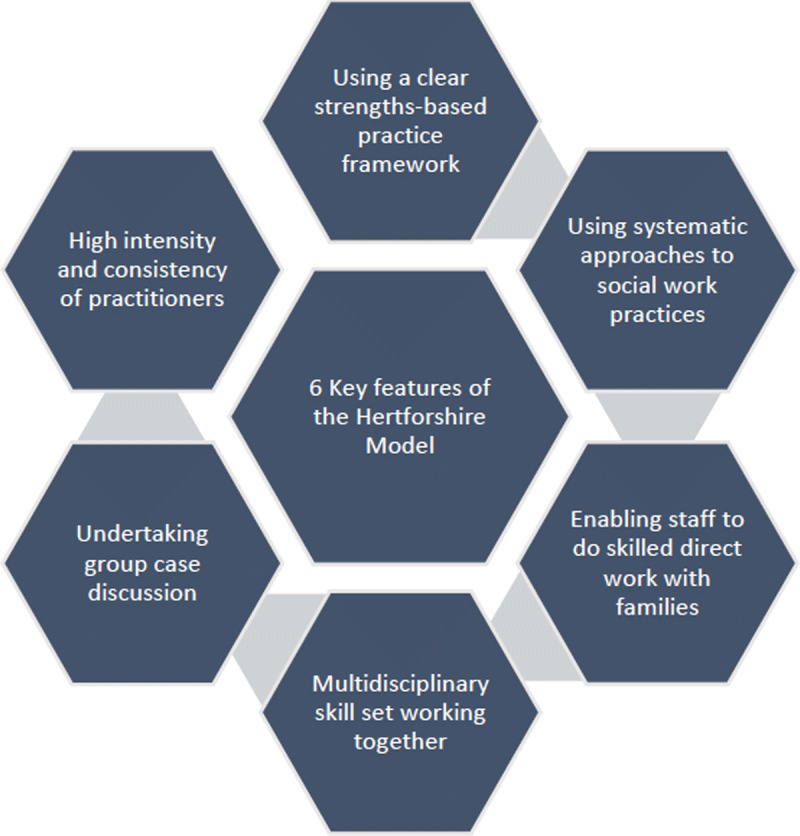
The key parts of Hertfordshire model.

The Oxfordshire model (FSP) has built on the experience of the Hertfordshire approach to family safeguarding by taking a multi-agency approach, employing motivational interviewing and providing help for both adult and child members (Figure 1).

FSP also draws on the experience of Hampshire County Council, which has successfully integrated assessment and intervention functions within the same teams and improved administrative support to social workers. FSP aims to help parents address the challenges that undermine their capacity to provide safe parenting, and social workers and others in the team develop a plan with families that builds on their strengths. The model places a much stronger emphasis on building and maintaining relationships, both within the family and between staff and family members. These aspects of the model should help children remain at home with less direct intervention from social services and are part of aims of the model. The aims of the FSP model, as described by OCC, are set out in **Box 1**.

**Box 1: Aims of the FSP model**

- Rebalancing the safeguarding system to help keep more families together where this can be achieved safely
- Developing a system based on working with family’s strengths
- Encouraging professional relationships that are empowering and help families to make positive changes
- Ensuring interventions are timely, focused and intense (no longer than they need to be)
- Working in a holistic, integrated way by supporting parents to address problems that impact on their ability to look after their children
- Reducing demand for children safeguarding services safely and appropriately

A theory of change was not explicitly generated by OCC when they developed FSP to be tested in such an evaluation, although the Hertfordshire model on which FSP is based specified a theory of change (see [20] for reference). However, the underlying rationale for this model is that the new programme will strengthen relationships between families and social workers and improve coordination of care for the whole family, which in turn will help to create a safe home environment so that children may be kept together with their families and the demand for child safeguarding services is reduced.

FSP has several key components, which build on and enhance the previous child safeguarding service.

#### Increased staffing with the aim of more contact time with families

An increase in the number of teams from 11 to 17 across OCC. This will reduce the caseloads for the social workers (with a target of 15 children for each full-time social worker, compared with 30 in the previous service) and reduce the supervisory caseload of team managers. Social workers will now undertake both the assessment of a family and later support, thus providing continuity during the process and time for closer engagement with the family.

#### Integrated multi-disciplinary teams

The new service is delivered by small multi-agency teams who are trained in a range of effective interventions. Each team will share three adult workers who provide specialist expertise in domestic abuse, substance misuse and mental health. These services are commissioned from established voluntary organisations, which specialise in the relevant disciplines: Elmore Community Service (domestic abuse); Turning Point Drugs and Alcohol Recovery Service (substance misuse); Oxfordshire MIND (mental health). All agencies working with the family contribute to planning, information sharing and the management of risk. Also, there are a set of deliverable outcomes agreed between OCC and the voluntary organisations to ensure that the aims of services are achieved. Lastly, the teams will also develop integrated working with local Housing departments in the city of Oxford and local district councils, delivering targeted support to families who are homeless or in vulnerable housing.

#### Motivational Interviewing

Team members will be trained to a high degree of skill in motivational interviewing, which provides a much stronger focus on building a supportive relationship with family members. Motivational interviewing is an evidence-based therapeutic interviewing method aimed at engaging parents to address challenges in their lives and foster more caring and responsible parenting. Wherever possible, the aim of these teams will be to work with parents to support them to change their behaviours, engage with support and to keep children safe at home.

#### Improved communication and integration of information

An integrated family workbook has been designed to integrate information about each family into one central record. This electronic database will support multi-agency teams in making critical decisions, such as when a family no longer poses any risk to the child. The workbook will replace existing recording requirements such as case notes, chronologies and assessments reports that are repetitive and time consuming. A key aim of the family workbook is to reduce bureaucracy and increase time for more purposeful direct work with families.

#### Integration of assessment and intervention functions

The previous child safeguarding service had separate child and family assessment teams and teams that were providing interventions. This led to a change in social worker at the end of the assessment, resulting in bureaucratic delays linked to transfer processes and, crucially, a disruption of the relationship of trust created between worker and family. The FSP teams work on the basis that one worker is allocated to undertake a shorter more intensive assessment and continues to deliver the agreed plan, accessing and co-ordinating the family safeguarding workbook and interventions to facilitate a timely and positive outcome for the child.

#### Governance of FSP

The progress of the implementation, monthly performance and benefits realisation are monitored by the Directorate Leadership Team and reported regularly to the Chief Executive and Cabinet who are part of OCC. The development and embedding of the model are supported by the Family Solutions Plus Partnership Board, which brings together the partnership of FSP providers and wider multi-agency stakeholders, chaired by the Deputy Director of Children’s Social Care, the project’s sponsor. The role of the Board is to oversee the implementation, support good outcomes, contribute to and receive the evaluation, and to seek means of ensuring the service is sustainable in the long-term.

## Methods and Analysis

### Study aims

The proposed programme of evaluation aims to understand the impact of the transition from a child-focussed safeguarding approach to one that focusses on supporting the whole family. Our principal research questions are as follows:

- What has been the impact on time children spend in contact with services and the nature of the support provided?
- What has been the impact on the experiences of children and families?
- What has been the impact on the experiences of staff?
- What impact has the intervention had on outcomes for children, in particular whether they remain in the family or needed to be cared for outside the home?

### Study design

We aim to measure the impact through analysis of routine data collected by OCC which will be complemented by an exploration of the experiences of different stakeholders through qualitative methodology.

Our evaluation will monitor changes through a baseline period: the launch of the new service in November 2020 until full implementation in April 2021. The evaluation will assess a number of process and outcome variables. Having consulted several evaluation frameworks, our approach broadly follows that laid out by Pawson and Tilley (1997) in their CMO model, which seeks to examine the relationships between context, mechanisms and outcomes [22]. Information from historical data held by OCC from at least the three years prior to the start of the new service in November 2020, to account for the impact of the COVID-19 pandemic, will be used to determine historical baseline levels and trajectories. We will also examine data from communities in other local authorities in England with comparable socio-demographic characteristics. We will monitor the impact on families, staff and services for a period of up to two years from full implementation of the new services in April 2021. In the present paper, we focus on the studies that will be carried out between November 2020 and March 2023. Further follow up studies in later years will follow the same format as those described here.

We will also review the data collected by providers of services commissioned by OCC as this becomes available. Organisations providing support for mental health problems, for instance, will be collecting their own outcome data, which may, in the longer-term, offer an additional perspective on the acceptability and impact of the new service.

### Study setting and population

Approximately 146,123 children live in the county of Oxfordshire as of 2019, comprising 21% of the county’s total population. Of these, 27,877 children are living in poverty [23]. In 2020, there were 22,261 contacts made to social care services in relation to child welfare and 376 workers in children’s social care. For comparison, statistical neighbours had 14% less contact made [24].

There have been 4,833 domestic abuse incidents where children were present in the year to December 2020 in Oxfordshire. In this period 1,365 children were subject of a child in need plan, 525 children were subject of a child protection plan and there were 771 placed in care either temporarily or permanently. There is a large variation in socio-economic deprivation and ethnic background in Oxfordshire compared to neighbouring counties [25].

### Assessing the impact of the new service on the experience of families

We will use a number of methods to assess the impact of the new services on the experiences of children and families and the consistency and standard of service provided to them.

#### The experience of parents

We aim to recruit 15 families annually from the start of the model implementation and two years post implementation of FSP. Families will be recruited with the help of FSP staff. If the staff receive consent from parents to take part, then a member of the research team will contact them to recruit them for interview. Interviews will be conducted either face-to-face or online depending on the availability and preference of the individual. Participants will be asked about their experiences with the new services and, where relevant, with the previous child safeguarding services.

We will employ individual interviews to ask parents to reflect on their relationship with their social worker: (i) feeling included in plans; (ii) feeling understood; (iii) understanding the next steps and the direction of the plan; (iv) understanding the outcomes and expectations; (v) whether they feel that commitments made by the social workers and others were kept.

#### The experience of children

The experience of children within the service is obviously the most critical reflection of the impact of the programme, both in the short and long-term. Due to the sensitivity and vulnerability of children within the safeguarding programme, interviews will initially be carried out by OCC staff who are already known to the children, but with researchers present in the interviews. This is in accordance with procedures followed in similar studies where vulnerable children have been interviewed for research in a safeguarding setting [26,27,28]. For example, in a study conducted by Woolfson and colleagues (2010), children were recruited through their social worker and interviewed by a practitioner with a research assistant present [28]. There were also protocols in place to offer support if a child became emotionally distressed during or following the interview [28]. We will seek to interview 15 children in the first set of interviews, with potentially larger numbers in future years. In these interviews, we will seek to ascertain whether their daily lives and future prospects of safety and stability have increased.

### Assessing the impact of the new service on the workload and experience of staff

In order to assess the impact of new service on staff and their experiences with the new model we will evaluate their experiences through focus group interviews.

We aim to recruit 1–3 focus groups with 6–8 staff per group, with different level of experience and status to provide a wider understanding. The participants will be recruited with the help of FSP head of services, they will inform the staff about the study and invite them to participate. The researchers will provide them with the different dates to join the focus group discussion either online or face-to-face depending on staff preference and availability.

We will use these interviews to explore staff views of: (i) preparation and training for the new service; (ii) overall views of the impact of the new services; (iii) effect of new services on their relationship with the families; (iv) impact on their work satisfaction and working conditions; (v) their views of the impact on wider council and partner services.

### The impact of FSP on services and outcomes for children and families

Evaluation of longer-term outcomes and the impact on services will be primarily achieved by analysis of quantitative data held by OCC, though supplemented by information derived from staff and family interviews. This data will be accessed through the OCC database and will be used to monitor the trends at the baseline and regularly every six months as a follow-up. We assume that changes in the delivery of services will be most apparent early on, with changes in outcomes, such as the numbers of children in care, only being apparent in the longer-term. We will monitor the evolution of the programme, examining such features as the caseload of social workers, the number of families within each team, the involvement of wider services for mental health, domestic abuse and substance misuse and other features of the FSP programme.

We will primarily focus on the length of time children spend within the service after initial assessment and the final outcome, initially over a one-year period and then following up longer-term. We will, for instance, examine the number of families subject to child protection orders and how long they remain in this position before a resolution is achieved. The most critical outcome indicators are the number of children successfully remaining at home and discharged from the service and the number who ultimately need to be cared for outside their family. We will also examine the impact of FSP for families with different constellations of risk factors, assessing for instance whether the impact of the approach varies according to the nature and extent of concurrent problems such as substance abuse and mental health problems. Table I provides a list of the key variables that we will be extracting from the full OCC database.

**Table I:** Principal variables supporting evaluation.


DEMOGRAPHICS

Age of child

Number of siblings

Ethnicity

Deprivation index

**FAMILY RISK FACTORS**

Domestic abuse or violence

Substance abuse

Mental health problems: child

Mental health problems: family

Learning disabilities

Number of previous assessments

Number of previous intervention plans

**FAMILY INTERVENTIONS**

Domestic abuse support

Substance abuse support

Mental health support

**CARE OF CHILDREN WITHIN SERVICE**

Length of time within service

Length of time from assessment to discharge



Number of children on Child in Need Plan

Length of time in Child in Need Plan

Number children on Child in Need Plan > 9m



Number of children on Child Protection Plans

Length of time in Child Protection Plan

Number children on Child Protection Plan for > 12 months



Number of children looked after outside the home (placed in care)

Nature of care arrangement

Number of children < 13 placed in care

Length of time in care before permanent solution

**EVENTUAL OUTCOMES (INITIALLY AT 1 YEAR)**

Number of children returning home

Number of children cared for temporarily but then returning home

Number of children requiring permanent care

Nature of permanent care


### Statistical analysis

Descriptive statistics will be used to summarise the data available and explore potential temporal trends and other associations. Initial evaluations will examine the impact of FSP by looking at changes over time within the OCC service. We will compare the experience and outcomes of children and families assessed in the two years before the programme began with those subsequently entering the programme.

We also plan to examine the impact of FSP using national data by comparing OCC with neighbouring services. This approach models the expected difference between the areas where the new service is delivered and control areas, where the intervention is not taking place, that are deemed to have similar socio-economic demographic characteristics (commonly referred to as ‘statistical neighbours’), based on information pre-intervention. We can therefore examine whether there are changes seen in the OCC programme that have not been seen in neighbouring services, thus complementing and extending the within service evaluation. The main advantage for using this approach is that it cancels other potential effects due to seasonality or systematic changes to recording and/or care systems, as these affect OCC and its statistical neighbours in similar ways.

We will use graphical methods based on Splines to present these expected differences and plot them against observed differences for visual comparison. Formal statistical evaluation of the new service will be based on observed changes to the expected estimates using generalised linear (mixed) models (with robust standard errors to adjust and account for autocorrelation) or other alternative approaches depending on the type of data (e.g. Poisson models for rates).

### Approach to qualitative data collection and analysis

Both individual interviews and focus group discussions will be digitally recorded and transcribed verbatim. Data analysis will follow thematic analysis using constant comparison to provide a rigorous approach to the emerging themes.

Each focus group and interview will be openly coded first (examining, comparing, conceptualising and categorising data) before being more consistently coded, a process by which codes are grouped into larger codes. These codes are then used to generate the themes. Data is constantly reviewed to see which themes they best fit with. All included data will be anonymised. Any identifiable data will also be removed or changed. Data will be held in a secure location at the University of Oxford for at least three years.

### Potential expansion of core evaluation programme

The research programme described above will provide valuable information on the impact of the programme on the families, staff and the core OCC safeguarding services. There is also potential, resources and access permitting, to expand the research programme in a number of respects. We are currently in the process of examining the potential for data linkage with partner organisations and service providers. If these can be achieved, we will have access to further secondary data from partner agencies on the effectiveness of the intervention. There is potential for further analysis of the following information, where we plan to map the police and health data pre- and post-implementation of FSP to examine the impact on partner services.

### Potential economic evaluation

OCC will be assessing costs and benefits of FSP within the overall service budgets and framework. However, we will also look to explore the cost-effectiveness of FSP further, by considering the caseload costs per plan, staffing costs and placement costs per service day, from which a cost to services per service day could be calculated. Pairing this with data already collected on the length of time children spend in the service, we would be able to make an assessment of the value of FSP. Cost-effectiveness analyses such as these are commonly conducted in these types of studies [for example, see 29]. Additionally, if we can track families’ use of the NHS and other services, then it may also be possible in time to consider the wider economic costs and benefits on services more generally. Downstream use of services by the families could potentially be extracted from routinely collected data and valued using unit costs available from the financial teams of the service provider or national average estimates [30].

### Ethics and dissemination

The project is run by OCC and it is approved by their governance procedures. Additional studies will be approved by Oxford University Ethics.

## Discussion

This paper describes the design of a mixed methods evaluation of children’s safeguarding services in the county of Oxfordshire. The evaluation is designed to provide a holistic understanding of the progress and impact of FSP. By publishing the protocol for this service evaluation, we make our methodological choices explicit and transparent, and add to the literature on service evaluation in social care. The evaluation of FSP also provides an opportunity to examine the benefits of integration in social care, as the programme brings a much stronger focus on the family voice in services and aims to coordinate and provide multiple services in a single, holistic programme of support. The integration of services (multi-agency approach) is an improvement in children’s safeguarding services, while working with adults is a unique aspect of the model.

Although we have specified as much as we can in advance, we recognise the importance of using a flexible methodology that can accommodate the inevitable changes and unexpected events that will occur in any large-scale system transformation. The Medical Research Council guidance on complex intervention trials calls for standardisation but also recognises the need to ‘describe the constant and variable components of a replicable intervention’ [31]. In this light, process and outcome evaluation of social care interventions allows for the exploration of intervention delivery and impact across diverse settings to understand how interventions are adapted to a local context.

### Strengths and limitations

The complex nature of the family safeguarding intervention poses a number of challenges for evaluation. Some of the most important in our view are: (i) the precise timing of the changes, given the need for a transition period, cannot be fixed with any certainty; (ii) we recognise that multiple people and agencies are involved in these changes, but at present we only have the resources to examine the views of families and council staff; (iii) interviewing very young children and infants will not be feasible due to their developmental stage and verbal communication capability; (iv) we cannot be certain, due to continuing negotiations on the availability and governance of data, whether we can assess impact on the wider use of health and other services.

Despite these limitations, we believe that the proposed research has many strengths. The richness of the data being collected in this project and the mixed methods approach increases the likelihood that the findings will be informative and that we will be able to track the evolution of the programme over time. Moreover, the evaluation benefits from a very close collaboration between the research team, council staff and local partners in service delivery. This embeddedness in social care practice means the research is well placed to navigate the complexity of children’s safeguarding services and ultimately, to bridge the divide between academic research and service delivery.

### Dissemination and impact

Data from the overall evaluation will be disseminated through various conduits including newsletters, community forums, and meetings with partners, academic publications and conference presentations to national and international audiences. We hope that the evaluation findings will be a valuable resource for other services seeking to develop family safeguarding models.

In addition to the specific findings, we hope that our evaluation and wider work within OCC will provide commissioners and social care leaders with a better understanding of both the challenges and potential of service redesign and increased collaboration between children’s social care and partnership services. We also believe that the close collaboration between social services and researchers in developing a framework and programme of evaluation studies may also encourage other such collaborations in the future.

The initiatives taken by Oxfordshire and other county councils represent a considerable departure from current models of child safeguarding towards a more collaborative and effective family-based model which should provide better outcomes for children in both the short and longer-term. Examining its impact will be critical to both developing and sustaining the new approach and to understanding how best to support vulnerable children and their parents.

## Data Accessibility Statements

Data related to the study is available upon request and with the permission of Oxfordshire County Council.

## Footnotes

Ofsted is the Office for Standards in Education, Children’s Services and Skills. They inspect services providing education and skills for learners of all ages. Also they inspect and regulate services that care for children and young people [16].
